# Visualizing the Domino-Like Prepore-to-Pore Transition of Streptolysin O by High-Speed AFM

**DOI:** 10.1007/s00232-022-00261-x

**Published:** 2022-08-18

**Authors:** Hirotaka Ariyama

**Affiliations:** Nano Life Science Institute (WPI-NanoLSI), Kanazawa University, Kakuma-machi, Kanazawa, Ishikawa 920-1192 Japan

**Keywords:** Pore-forming proteins, Atomic force microscopy, Pore formation, Transmembrane β-hairpins, Propagation

## Abstract

Pore-forming proteins (PFPs) are produced by various organisms, including pathogenic bacteria, and form pores within the target cell membrane. Streptolysin O (SLO) is a PFP produced by *Streptococcus pyogenes* and forms high-order oligomers on the membrane surface. In this prepore state, multiple *α*-helices in domain 3 of each subunit exist as unfolded structures and transiently interact with each other. They subsequently transition into transmembrane β-hairpins (TMHs) and form pores with diameters of 20–30 nm. However, in this pore formation process, the trigger of the transition in a subunit and collaboration between subunits remains elusive. Here, I observed the dynamic pore formation process using high-speed atomic force microscopy. During the oligomer transition process, each subunit was sequentially inserted into the membrane, propagating along the oligomer in a domino-like fashion (chain reaction). This process also occurred on hybrid oligomers containing wildtype and mutant subunits, which cannot insert into the membrane because of an introduced disulfide bond. Furthermore, propagation still occurred when an excessive force was added to hybrid oligomers in the prepore state. Based on the observed chain reactions, I estimate the free energies and forces that trigger the transition in a subunit. Furthermore, I hypothesize that the collaboration between subunits is related to the structure of their TMH regions and interactions between TMH–TMH and TMH–lipid molecules.

**Graphical Abstract:**

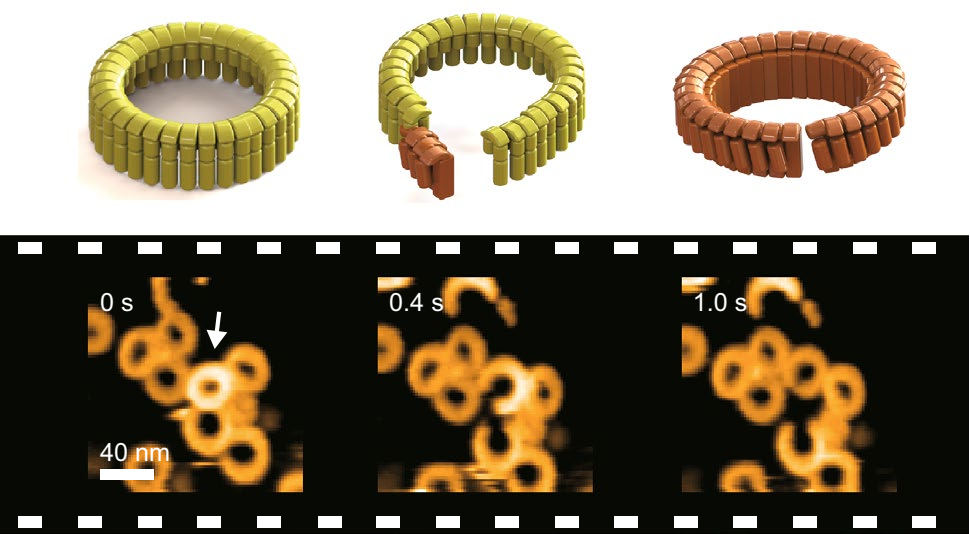

**Supplementary Information:**

The online version contains supplementary material available at 10.1007/s00232-022-00261-x.

## Introduction

Many common pathogenic bacteria secrete pore-forming proteins (PFPs) as virulence factors that cause fever, septic shock, and other adverse effects (Gilbert et al. 2014; Los et al. 2013; Peraro and Van Der Goot 2016). In addition, PFPs such as the membrane attack complex and perforin proteins (MACPFs) are used for immune defense by animals. The cholesterol-dependent cytolysins (CDCs) expressed by Gram-positive bacteria and some Gram-negative bacteria constitute a large PFP subfamily that includes streptolysin O (SLO) and perfringolysin O (PFO) (Hotze et al. 2013; Hotze and Tweten 2012). CDCs are secreted as soluble monomers that bind to the target cholesterol-containing cell membrane, oligomerize onto it, and form pores through it. This pore state causes cellular ion imbalance and eventually host cell death. Therefore, understanding the molecular mechanisms underlying the CDC pore formation process is crucial for developing therapeutic strategies for CDC-induced diseases.

**Fig. 1:**
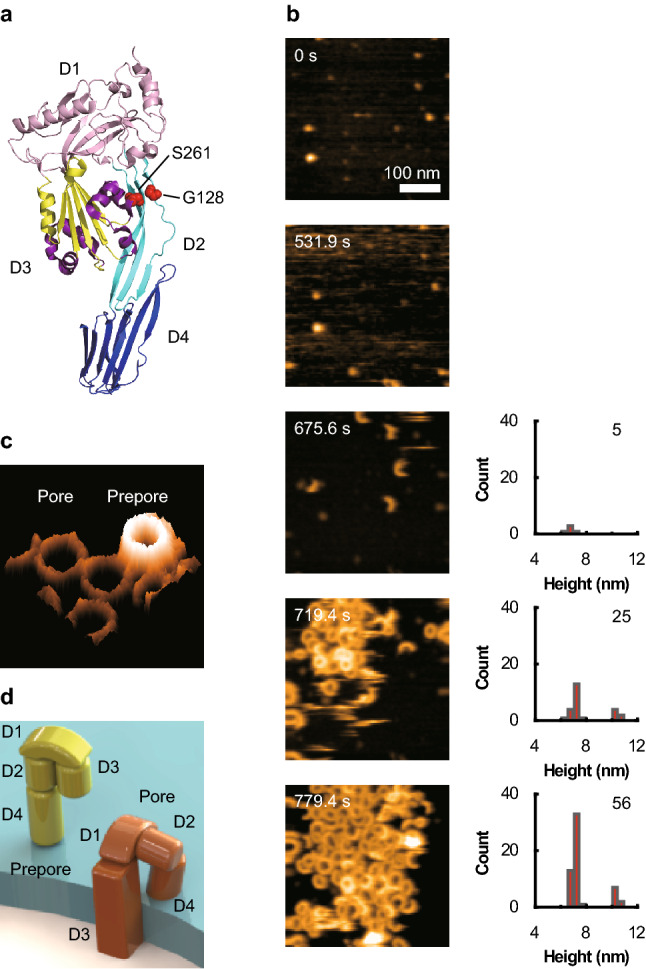
The three-dimensional structure, successive AFM images, and time-dependent height distribution of SLO. **a** The three-dimensional structure of SLO. SLO is composed of D1–4 domains, with the TMH1 and TMH2 of D3 shown in purple (PDB code: 4HSC). After interacting with the membrane, the helical regions convert to β-strands and insert into the membrane. In the SLO (C/A)–SS mutant, cysteines (red squares) were introduced into D2 and D3 (S261C and G128C). **b** Successive AFM images of SLO interacting with the membrane and the height distribution of oligomers on the membrane. SLO was added to the AFM liquid cell at a final concentration of 0.2 μM. SLO binding to the membrane, oligomer formation, and oligomer insertion into the membrane were then observed. The numbers in the height distribution denote the number of oligomers observed. **c** AFM image of oligomers on the membrane. Oligomers with a height of 10–12 nm are in the prepore state, and oligomers with a height of 6–8 nm are in the pore state. In EM data from Sekiya et al., the height of oligomers in the prepore state was 10.9 nm and in the pore state was 6.3 nm (Sekiya et al. 2007), which is consistent with my data. **d** A model of a subunit in both the prepore and pore state. The prepore state (yellow) refers to a state in which subunits in the solution bind to the membrane and form high-order oligomers. In the pore state (brown), these oligomers undergo a structural change, inserting the TMH regions into the membrane and forming pores

The CDCs share a 40%–80% amino acid sequence identity. The crystal structure of several CDCs in soluble form has revealed four domains, D1–D4, and a key region for membrane recognition(Bourdeau et al. 2009; Köster et al. 2014; Lawrence et al. 2015; Polekhina et al. 2005; Rossjohn et al. 1997, 2007; Xu et al. 2010) (Fig. 1a). The oligomerized CDCs have been imaged by electron microscopy and atomic force microscopy (AFM), revealing a structure of arc- or ring-shaped oligomers containing 30–50 subunits (Czajkowsky et al. 2004; Sekiya et al. 2007; van Pee et al. 2017). These oligomers are formed through the interactions of the D1 and D3 domains of different subunits and have been observed to have different heights: the higher (prepore) and lower (pore) states. Biochemical studies of CDC mutants have identified a region of transmembrane amphipathic β-hairpins (TMHs) that transitions from *α*-helices to β-hairpins (Shepard et al. 1998). Based on previous studies, the following model has been proposed for the pore formation process. First, the CDC molecule recognizes cholesterol at a conserved Thr–Leu pair known as the cholesterol recognition/binding motif (CRM) in Loop 1 of D4 (Farrand et al. 2010; Feil et al. 2014). Upon the D4–cholesterol interaction, D3 loses contact with the rest of the CDC, causing the release of the membrane insertion regions (TMH1 and TMH2) from the body of D3. According to disulfide-bond scanning, TMH regions are not *α*-helices and β-hairpins, partially unfolded, move dynamically, and transiently touch the same regions of an adjacent subunit without stable hydrogen bonds (Sato et al. 2013). Finally, these regions are converted into β-hairpins in all oligomer subunits, forming a β-barrel pore with a 20–30 nm diameter.

Despite recent advances, two questions regarding pore formation remain to be answered. What triggers the prepore-to-pore transition in a subunit? And how do the subunits collaborate during the insertion process? First, two molecular models have been proposed as concepts for understanding triggering. In the first E–K pair model, a Glu–Lys (E–K) pairing between subunits is formed after tilting of D1–3, causing destabilization of TMH–D1/2 interactions and releasing TMH regions (Wade et al. 2015). In this model, D2 only rotates around the D2–D4 linkage but does not deform. In the alternate lipid model, contact between lipids and TMH regions attracts them to the membrane, causing D2 to buckle and the regions to insert into the membrane (Czajkowsky et al. 2015). However, since there has been no direct observation of the pore formation process, which model is correct remains unclear. Second, as an approach to understanding the collaboration between subunits, hybrid oligomers consisting of the wildtype (WT) and mutant subunits that cannot insert into the membrane have been used. Hybrid oligomers were observed to form pores in these studies, indicating that there is some collaboration of subunits in the pore formation (Hotze et al. 2002; Leung et al. 2014). However, similar to the first issue, because no study has directly observed the dynamic insertion process, details of the collaboration remain unclear.

Recently, PFPs have been observed using high-speed AFM (HS-AFM), which can help visualize the dynamic action of biomolecules (Leung et al. 2014; Ruan et al. 2016; Sriwimol et al. 2015; Yilmaz et al. 2013; Yilmaz and Kobayashi 2015). However, pore formation has yet to be visualized at high resolution. In this study, I performed HS-AFM imaging of the dynamic process of pore formation by SLO produced by *Streptococcus pyogenes* and an SLO mutant with a disulfide bond. My approach enables me to estimate free energies and forces that trigger the prepore-to-pore transition and to infer molecular transition models. Furthermore, it reveals how the subunits collaborate to insert into the membrane and that their collaboration is essential for pore formation. I report the first direct observation of subunit collaboration and suggest that critical components of this collaboration are the structure of TMH regions and the interactions between TMH–TMH and TMH–lipid molecules.

## Results

### Prepore-To-Pore Oligomer Transition and Oligomer Structural Changes in the Pore State

**Fig. 2:**
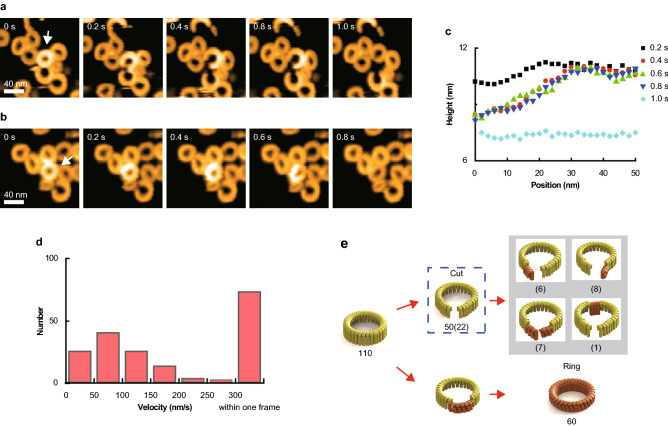
AFM analysis of the prepore-to-pore transition. **a** The prepore-to-pore transition was observed by adding SLO to the AFM liquid cell at a final concentration of 0.2 μM. An oligomer’s height on the membrane was observed to change from 10–12 to 6–8 nm. In this case, a ring-shaped oligomer in the prepore state was cut. **b** Using the same experimental conditions as (**a**), the change in oligomer height was observed. However, in this case, a ring-shaped oligomer in the prepore state was not cut. **c** The time-dependent change in oligomer height (indicated by the white arrow in (**a**)) on the membrane. The position is provided as the distance between the two ends of the arc-shaped oligomer. At 0.2 s, all subunits were in the prepore state. At 1.0 s, all subunits were in the pore state. There was a 30 nm height gradient from the one end at 0.4, 0.6, and 0.8 s. These data indicate that the subunits at one end were in the pore state and the prepore state at the other end, with subunits in between partially inserted into the membrane. **d** The propagation velocity along oligomers during the prepore-to-pore transition. **e** The classification of the propagation process during the prepore-to-pore transition. Numbers indicate the number of oligomers observed. The numbers in parentheses denote the number of oligomers observed for multiple frames

In the AFM liquid cell-containing Buffer A, SLO was added to a final 0.2 μM concentration. On the AFM sample stage, lipid bilayers composed of a 40% 1,2-dioleoyl-*sn*-glycero-3-phosphocholine (DOPC) and 60% cholesterol (Chol) mixture were covered (Fig. 1b). Only the membrane surface without SLO was observed for 7–8 min after the addition, then spike-like noise running in the X-direction appeared. Short arc-shaped oligomers then appeared, soon followed by the appearance of ring-shaped oligomers. The number of ring- and arc-shaped complexes quickly increased, covering nearly the entire membrane surface. Oligomers formed at this stage were classified into two groups according to their height: those 10–12 nm high constituted the higher (prepore) state, and those 6–8 nm high made up the lower (pore) state (Fig. 1b–d). Oligomers in the prepore state diffused faster than in the pore state, while simultaneously changing their shape. While the number of oligomers increased on the membrane, the prepore-to-pore transition of oligomers was observed. Most oligomers in the prepore state quickly transitioned into the pore state within a frame duration (200–300 ms). However, in some cases, this transition occurred more slowly (Fig. 2a, b; Movies S1 and S2) and in two distinct manners.

In the first case (Fig. 2a; Movie S1), a ring was cut at one position and its ends opened outward, creating an arc-shaped oligomer, and one end region of the arc-shaped oligomer quickly transitioned into the lower state. This transition propagated along the oligomer in one direction like a chain reaction. In the other case (Fig. 2b; Movie 2), the entire ring was in the higher state before a part of the ring transitioned into the lower state without the ring being cut and the transition propagated along the oligomer. The time-dependent height profiles along the oligomer undergoing height transition are shown in Fig. 2c. It is evident that all parts of the oligomer ultimately shifted to the lower state. During the propagation of height change, there is a border region in which height is intermediate between the lower and higher states, indicating that some subunits were only partially inserted into the membrane, rather than fully. An analysis of height change propagation for oligomers that did not transition within the span of a frame (200–300 ms) found a distribution of propagation velocities peaking at around 50–100 nm/s (Fig. 2d), corresponding to the transition of 30–60 subunits/s. Among the 110 ring-shaped oligomers analyzed, 50 were observed to be cut, while the rest underwent the prepore-to-pore transition without being cut. Among the 50 cut rings, 28 transitioned into the lower state within the span of a frame, while the remaining 22 showed propagation of height transitions along the oligomers in one of the four ways (Fig. 2e, right panel). In most cases, this propagation started from the cut region. When only one end of the cut region underwent height change transition, this propagation occurred in one direction, starting from the end showing height transition. Although TMHs are tilted at 20° to the membrane perpendicular, the probabilities of clockwise (0.45) and counterclockwise (0.55) propagation were similar. When both ends underwent height change transition, the propagation was bidirectional, starting at each end.

Understanding the molecular mechanism underlying the domino-like propagation is crucial. Disulfide scanning has shown that TMH regions in D3 of a subunit in the prepore state are partially unfolded, but not the *α*-helices and β-strands (Sato et al. 2013). Furthermore, TMH regions are free to interact transiently with the same regions of an adjacent subunit. When TMH regions insert into the membrane, the interaction between their hydrophobic amino acids and lipid molecules induces a structural change in the part of TMH regions in contact with the membrane into a partial β-strand. That part of TMH regions then experiences a stronger attraction to the membrane through interactions with its lipid molecules, converting it into a complete β-strand and inserting it into the membrane. On the other hand, TMH regions of the adjacent subunit are similarly attracted toward the membrane through TMH–TMH interactions, where they insert into the membrane and convert into complete β-strands. Thus, the reaction propagates along the oligomer, leading to the eventual transition of all subunits from the prepore to the pore state.

Moreover, protrusions from the membrane at the central pore of oligomers were observed in the prepore state. During the prepore-to-pore transition of ring-shaped oligomers, the height of central pores decreased from 2–3 nm to − 1 or 1 nm in 0.2–3 s (Fig. S1). While the height of the central pore has not been previously explored, examining the height is important for elucidating the transition mechanism at the molecular level. Therefore, I analyzed the height of oligomers in the prepore and pore states. However, it was particularly challenging to analyze this parameter in the context of the above experiments since the entire membrane was covered with oligomers, and its surface was not directly observed. Therefore, I performed additional experiments, in which I washed away unattached SLO molecules ~ 3 min after its addition. Consequently, the height of the central pore of oligomers from the membrane in the prepore state was determined to be 3.3 nm and − 1.3 nm or 0.4 nm in the pore state (Fig. S2). My findings in the prepore state suggest that TMH regions in D3 may pull-up lipid molecules, while the height of − 1.3 nm in the pore state suggests that some lipid molecules of the central pore had been removed from the membrane.

In addition, the experiments conducted after membrane washing also provided some intriguing results, unrelated to the pore transition process. I observed that small arc- or ring-shaped oligomers in the pore state diffused in the membrane and bound to each other to form a ring. During this process, the gradual growth of oligomers where one subunit binds to other subunits was not observed (Fig. S3; Movie S3). Moreover, the diameter of oligomers 25 min after removal of unattached SLO was identical to after 5 min (Fig. S4). Even after repeat bindings, the number of subunits in oligomers remained constant. These data indicate that there is an optimal number of subunits to form a ring, determined by intersubunit interactions. Furthermore, in addition to the formation of high-order oligomers, the self-association and dissociation of oligomers in the pore state were observed (Fig. S5). Thus, this experiment elucidated the diffusion and process of binding in the pore state, the optimal number of subunits, and how oligomers change over time. A similar diffusion of oligomers in the pore state and interactions between oligomers have been reported for other CDCs, indicating that oligomers are flexible and that the pores formed by several oligomers can function at the cell membrane (Podobnik et al. 2015; Ruan et al. 2016).

### Prepore-to-Pore Transition of Oligomers Consisting of WT and SLO (C/A)-SS Subunits

**Fig. 3:**
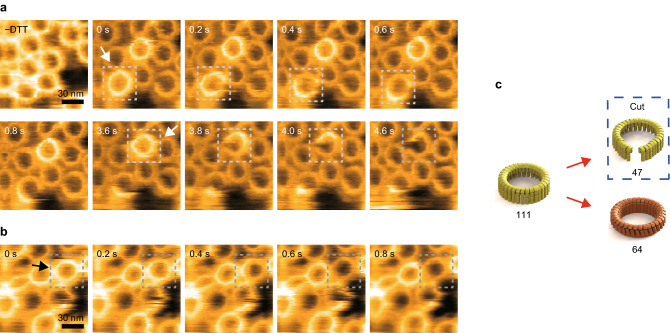
The prepore-to-pore transition of mutant oligomers with the addition of DTT. **a** Successive AFM images of mutant oligomers with DTT addition. After the mutant oligomers formed on the membrane, unattached mutant subunits were washed away, and DTT was added to the AFM liquid cell to a final concentration of 4–10 mM. In this case, the ring-shaped oligomers in the prepore state were cut (white arrows). **b** Using the same experimental conditions as (**a**), the change in oligomer height was observed. However, in this case, a ring-shaped oligomer in the prepore state was not cut (black arrow). **c** The dynamic prepore-to-pore transition process of ring-shaped oligomers. Of the 111 ring-shaped oligomers observed, 47 were cut during the transition

Because the prepore state is generally short-lived, mutants with a disulfide bond between D2 and D3 have often been used to observe CDCs in the prepore state (Hotze et al. 2001). Here, I prepared a similar mutant intending to observe more closely the prepore-to-pore transition. Furthermore, I intended to explore intersubunit cooperation by creating hybrid WT and mutant oligomers. The cysteine residue in D4 of WT SLO was substituted with alanine (C530A), and one cysteine was introduced into both D2 and D3 (S261C and G128C) to create the SLO (C/A)–SS mutant, which formed ring-shaped oligomers in the prepore state. As expected, mutant oligomers were never observed in the pore state. Upon the addition of dithiothreitol (DTT), 10%–20% of oligomers transitioned into the pore state within the initial 3 min. This proportion increased with time, eventually reaching 100% after 10 min. As previously observed with the WT protein, ring-shaped oligomers were often cut at one portion (0.2 s and 3.8 s in Fig. 3a; Movie S4), resulting in arc-shaped oligomers. In the arc-shaped oligomers, one end transitioned into the lower state. This transition propagated along the oligomer in one direction like a chain reaction. Among the 111 ring-shaped oligomers analyzed, 47 were observed to be cut, while the rest underwent the prepore-to-pore transition without being cut (Fig. 3). Next, I used a mixture of WT and mutant subunits at different ratios without DTT (Buffer B), adding it to the membrane and removing unattached SLO after a 3 min incubation. At a mixing ratio of 4:6 (WT:mutant), some of the hybrid oligomers were observed to be completely in the pore state ~ 10 min after washing the sample to the membrane. As with the mutant SLO in the presence of DTT, the prepore-to-pore transition propagated along the oligomer (Movie S5). During this transition, the intermediate state was also observed, where the height of a continuous segment within an oligomer was between the lower and higher states. The proportion of oligomers completely in the pore state increased with increasing time.

**Fig. 4:**
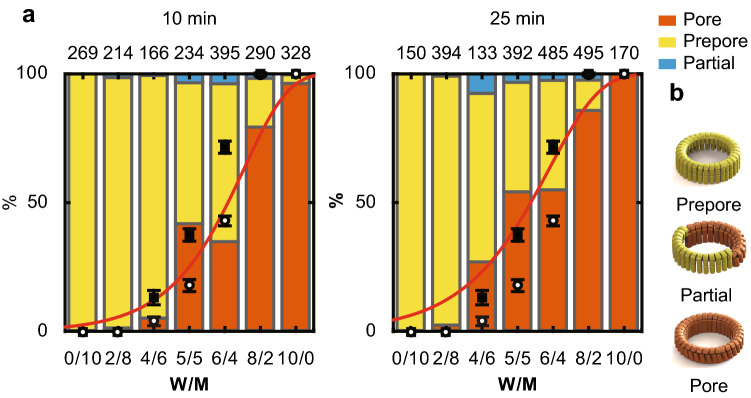
The proportion of oligomer structures mixed at different WT and mutant SLO subunit ratios. **a** The proportion of oligomer structures mixed at different WT and mutant SLO subunit (W/M) ratios. After forming hybrid oligomers with WT and mutant SLO subunits on the membrane, unattached SLO was removed. At 10 and 25 min after the addition of SLO, oligomers with different mixing ratios were observed and analyzed. The observed oligomers were classified by structure into prepore (yellow), partial (blue), and pore (brown). The numbers on the graph indicate the number of oligomers observed at each W/M ratio. The red lines denote fitting by Eqs. 1, 2, 3, and 6. The two ends of the graph are set to 0 and 1 in *r*. **b** The structure of oligomers consisting of WT and mutant subunits at different ratios. Oligomers consisting of subunits only in the prepore state are defined as prepore, in both the prepore and pore state as partial, and only in the complete pore state as pore

When the fraction of mutant subunits was increased, the number of oligomers completely in the pore state decreased (Fig. 4). Furthermore, oligomers in a partially transitioned state, with some subunits in the pore state and others in the prepore state, were rarely observed. Therefore, the prepore-to-pore transition occurred in all subunits.

My findings with hybrid oligomers indicate that WT subunits first transitioned from the prepore to pore state, and the prepore-to-pore transition of the subunits propagated along the oligomer. By determining the trigger for the first transition, I evaluated the free energies and forces involved in the prepore-to-pore transition in the discussion section.

Further, the height of the central pores of oligomers in the prepore and pore states was assessed (Fig. S2). I found that the height of the mutant subunit’s central pore in the prepore state was 0.5 nm, 2.8 nm lower than the WT subunit. TMH regions of SLO (C/A)-SS subunits cannot interact with the membrane because the movement of dissociation from D2 is restricted conformationally. Therefore, lipid molecules cannot be pulled up by TMH regions in D3. The results of this experiment are consistent with the view that TMH regions may interact with the membrane in the prepore state.

### Prepore-to-Pore Transition by an Excessive Force

**Fig. 5:**
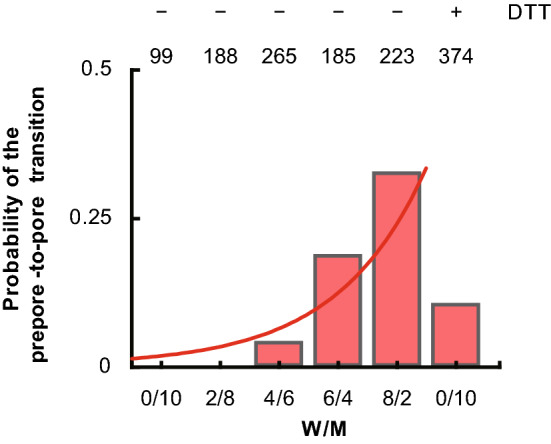
The probability of the prepore-to-pore transition induced by excessive force in oligomers consisting of different mixtures of WT and mutant SLO subunits. After the formation of oligomers on the membrane, unattached SLO was removed. During the observation of ring-shaped oligomers, excessive force was applied to one point on a ring-shaped oligomer with the cantilever tip. The effect of DTT was tested only on the oligomers entirely made up of mutant SLO subunits. Excessive force was applied up to 2 times per ring, and the number of oligomers inserted into the membrane was counted. The number on top of each bar indicates the number of times excessive force was applied. The red line denotes fitting by Eqs. 1, 2, 3, and 6, representing *P*_pore_ multiplied by *N*_*y*_. The two ends of the graph are set to 0 and 1 in *r*

An earlier study applied 50–106 pN of force to PFO trapped in the prepore state by a disulfide bond using a cantilever tip to push them toward the membrane, resulting in their transition to the pore state (Czajkowsky et al. 2015). Here, I conducted a similar experiment with SLO (C/A)–SS oligomers using a similar level of excessive force (25–100 pN). In my experiment, the locus onto which an excessive force was applied was prespecified using a mouse pointer after obtaining AFM images. Because the period of force applied was much shorter (~ 40 μs) and the locus area smaller than in the earlier study, mutant oligomers never transitioned into the pore state. Next, I used hybrid oligomers consisting of WT and mutant subunits at different ratios. As described above, a fraction of the hybrid oligomers spontaneously transitioned into the pore state, while the rest remained in the prepore state. When I applied an excessive force to the oligomers containing ≥ 40% WT subunits, the transition to the pore state occurred (Fig. 5). While some oligomers immediately transitioned to the pore state when the excessive force was applied (Movie S6), in other oligomers, the transition propagated along them in a chain reaction (Movie S7). In addition, the transition probability increased with the increasing fraction of WT subunits. For oligomers consisting only of mutant subunits, an excessive force applied after the addition of DTT induced transition with a probability of 0.12 3–10 min after DTT addition. Based on this probability and Fig. 5, the fraction of disulfide bond-reduced subunits was estimated to be about ~ 50% 3–10 min after DTT addition. In this experiment, I hypothesized that as TMH regions in some subunits were forced close to the membrane, they interacted with the membrane and converted into β-strands. Consequently, the TMH regions of adjacent subunits also approached the membrane and strongly interacted with it due to intersubunit interactions.

## Discussion

CDC monomers bind to the membrane and form high-order oligomers. In the prepore state, the monomer–monomer association of oligomers occurs through D1–D1 and D3–D3 interactions (Tilley et al. 2005). In the D3–D3 interaction, the *π*–*π* interaction between Tyr and Phe plays a significant role. When monomers diffuse on the membrane surface, the *π*–*π* interaction induces the intersubunit attraction between β 1 in D3 of one monomer and β 4 in D3 of another monomer to form oligomers (Hotze et al. 2012; Ramachandran et al. 2004). Then, the *α*-helices of TMH regions are unfolded, converted into β-strands, and inserted into the membrane (Sato et al. 2013), forming transmembrane hairpins that associate with adjacent subunits in the membrane. Consequently, the TMH regions of all the subunits form β-barrel pores.

**Fig. 6:**
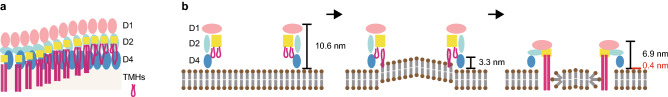
A model of TMH regions in the SLO subunits during the prepore-to-pore transition. **a** The model of TMH regions in SLO subunits with a conformational gradient during insertion. TMH regions in the prepore state start as partially unfolded *α*-helices with small β-strands and become complete β-hairpins in the pore state. In the intermediate state, subunits have several conformations. Part of the TMH regions in a subunit adjacent to subunits in the pore state insert into the membrane and refold into β-strands. However, in a subunit adjacent to subunits in the prepore state, TMH regions are partially unfolded *α*-helices. Although they may interact with the membrane, they are not inserted deep into it. The TMH regions in D3 are shown in purple, and the rest of D3 is shown in yellow. **b** A model of the prepore-to-pore transition. TMH regions interact with the membrane, and part of their unfolded *α*-helices convert into small β-strands. The membrane may be pulled up vertically by TMH regions, and the β-strands are pulled down toward the membrane. The remaining unfolded *α*-helices interact with the membrane. Finally, all unfolded *α*-helices convert to β-hairpins and are deeply inserted into the membrane. Due to the movement of the TMH regions, the lifted membrane goes down

In this study, the prepore-to-pore transition of WT SLO was observed at high spatiotemporal resolution (Fig. 2). In the initial prepore-to-pore transition stage, many ring-shaped oligomers were observed to be cut in one place, and the transition began at either one or both ends. This ring breakage indicates that the magnitude of the force by which TMHs are inserted into the membrane is sufficiently large to break all D1–D1 and D3–D3 intersubunit interactions. It is also possible that this breakage is due to the elimination of distortion in subunits. Incidentally, because oligomers in the prepore state are flexible, D1–D1 and D3–D3 intersubunit interactions may not be large and the sites of interaction between subunits may not be fixed. Following this initial process, it was observed that the last 7–10 subunits in the oligomer show a height gradient. Therefore, a corresponding conformational gradient related to height must exist (Fig. 6a). The conformations are likely to be complete β-strands inserted into the membrane, incomplete β-strands with unfolded *α*-helices, and partially unfolded *α*-helices. If the partially unfolded *α*-helices interact with lipid molecules, part of the *α*-helices may convert into β-strands. When the incomplete β-strands connected to unfolded *α*-helices are inserted further into the membrane, the interaction force likely pulls the unfolded *α*-helices downward, transforming them into β-strands. In adjacent subunits, partially unfolded *α*-helices with small β-strands are brought closer to the membrane through interactions between TMH regions with the membrane-inserting subunit. Upon contact with the membrane, the unfolded *α*-helices are believed to be gradually converted into β-strands. Thus, subunits act cooperatively to propagate membrane insertion processes along the oligomer in a chain reaction. From a different perspective, the large amount of energy acquired by one subunit through its hydrophobic interactions with lipid molecules is used to transition the adjacent subunit. The energy of the hydrophobic interaction per unit surface area of apolar compounds is 17–31 cal/mol/Å^2^, with an average of 25 cal/mol/Å^2^ (Blokzijl and Engberts 1993). Assuming that each monomer has a surface area of 1000 Å^2^, this would provide large hydrophobic interaction to support the prepore-to-pore transition.

Hybrid oligomers containing WT and mutant subunits were not observed to insert into the membrane when the fraction of mutant subunits was large. Conversely, a large fraction of WT subunits enabled the prepore-to-pore transition of hybrid oligomers, which propagated along the oligomer similar to WT-only oligomers. Based on the estimated molecular structure of the mutant subunit, they cannot contact the membrane without breaking their disulfide bonds. However, the TMH regions of mutant subunits are thought to be partially unfolded even though the disulfide bond suppresses their freedom of motion in D3. When the incomplete β-strands in a WT subunit are inserted into the membrane, its remaining unfolded *α*-helices interact with unfolded *α*-helices in an adjacent mutant subunit. Through this interaction, unfolded *α* -helices in the mutant subunit are pulled toward the membrane, promoting their transition to β-strands and initiating membrane insertion. Incidentally, all disulfide bonds may not be broken because their cleavage requires up to 66 kcal/mol.

Two models have been proposed for the molecular mechanism triggering the prepore-to-pore transition of one subunit. Wade et al. proposed the E–K pair model, in which E–K pairs created by the rotation of D1–3 form between subunits in the prepore state and cause more β 1 and β 4 in D3 to attract each other (Wade et al. 2015). Because the TMH regions are directly connected to β 1, they gain more freedom of movement as Glu and Lys approach. The energy barrier of 19 kcal/mol is then overcome throughout the oligomer by stabilization of more than − 1 kcal/mol per E–K pair between subunits. On the other hand, Czajkowsky et al. proposed the lipid model, in which the TMH regions interact with the lipid molecules that are pulled from the membrane, resulting in buckling of D2 and insertion of the TMH regions into the membrane (Czajkowsky et al. 2015). Their molecular dynamics (MD) simulations showed that destabilization of the TMH–D1/2 interface does not cause insertion of the TMH regions into the membrane.

According to the lipid model, force applied to the subunit toward the membrane causes buckling of D2, depending on the magnitude and the duration of the force. Based on a single barrier model, the probability of the prepore-to-pore transition in a subunit (*P*_pore_) is given by:

1 $${P}_{\mathrm{pore}}=1-\mathrm{exp}\left(-kt\right),$$

where

2 $$k=A\times \mathrm{exp}\left[-\Delta {G}_{0}/{k}_{\mathrm{B}}T\right]\times \mathrm{exp}\left[F{x}_{\upbeta }/{k}_{\mathrm{B}}T\right]$$

and *t* is a period under applied force, *A* is the attempt frequency, Δ*G*_0_ is the energy barrier height for a single subunit, *k*_B_ is Boltzmann’s constant, *T* is the temperature in kelvin, *F* is an applied force per subunit, and *x*_β_ is the reaction coordinate distance to the energy barrier peak from the minimum. To experimentally estimate the energy barrier height based on the force spectroscopy measurements using AFM, the force by the tip of the cantilever is added to the force pulling from the membrane. Therefore, *F* in Eq. 2 can be expressed as:

3 $$F={F}_{\mathrm{tip}}+{F}_{\mathrm{mem}}.$$

Since Czajkowsky et al. used the mutant subunit of PFO and TMHs were not pulled from the membrane, *F* was 50–106 pN provided only by the tip of the cantilever (*F*_mem_ = 0). Equations 1, 2, and 3 yield *x*_β_ = 5.1 Å and Δ*G*_0_ = 16.3 kcal/mol with the value of *A* = 10^7^/s reported in previous force spectroscopy studies (Yu et al. 2012).

My results on membrane protrusion in the central pore may be consistent with the lipid model. However, experiments on the probability of pore formation in hybrid oligomers without applying an excessive force can be explained by these two models. Under the E–K pair model, a WT subunit initiates the chain reaction if the neighboring subunits are also WT because the Glu and Lys of the mutant subunit are structurally less likely to interact with the adjacent Glu and Lys. The proximity of the E–K pair between subunits enables the TMH regions of that subunit to move more freely. I modeled the probability of this transition as dependent on the number of successive WT subunits in the oligomer. When five or six WT subunits are consecutive, the prepore-to-pore transition occurs (Fig. 4a). The probability of the prepore-to-pore transition in a subunit (*P*_pore_) can be expressed by equations similar to those above:

4 $${P}_{\mathrm{pore}}={P}_{\mathrm{con}}\times \left\{1-\mathrm{exp}\left(-kt\right)\right\}$$

5 $$k= A\times \mathrm{exp}\left[(-\Delta {G}_{0}+\Delta {G}_{\mathrm{E}-\mathrm{K}})/{k}_{\mathrm{B}}T\right]$$

where Δ*G*_E–K_ is the free energy contributed by the formation of the E–K pair and *P*_con_ is the probability of consecutive WT subunits in all oligomers. Because the chain reaction does not stop once a subunit inserts into the membrane, *P*_pore_ is considered the proportion of hybrid oligomers completely in the pore state (Fig. 4). Therefore, the transition probability of a subunit is the same for hybrid oligomers. Using the values of *A* = 10^7^/s, *t* = 600 s or 1500 s (10 min or 25 min), and *ΔG*_0_ = 19 kcal/mol, Eqs. 4 and 5 yield the energy per E–K pair of ~  − 1 kcal/mol (Table S1).

On the other hand, in the lipid model, assuming the fraction of the WT subunits is proportional to the height of the central pore, *F*_mem_ is considered proportional to the fraction of WT subunits. Therefore, it can be expressed as:

6 $${F}_{\mathrm{mem}}= {F}_{\mathrm{mem}1}\times r,$$

where *F*_mem1_ is the membrane force pulling the WT subunit and *r* is the fraction of WT subunits in hybrid oligomers. Using the values of *A* = 10^7^/s and *x*_β_ = 5.1 Å from Czajkowsky et al. and fitting the probability of pore formation with Eqs. 1, 2, 3, and 6, I obtained the values Δ*G*_0_ = 15.7 ± 0.6 kcal/mol (26.6 kBT at 25 °C) and *F* = 45.5 ± 12.3 pN at 10 min, and Δ*G*_0_ = 15.7 ± 0.4 kcal/mol (26.6 kBT) and *F* = 40.2 ± 9.2 pN at 25 min. These energies under both models are consistent with those reported in previous studies (Czajkowsky et al. 2015; Wade et al. 2015).

I applied an excessive force to hybrid oligomers that remained in the prepore state after the formation of oligomers. Propagation was observed along the oligomer during the transition. The period of force application was shorter than that used with PFO, and the area onto which the excessive force was applied was smaller (Czajkowsky et al. 2015). Consequently, the force was not sufficient to cause mutant subunits to insert into the membrane on their own. However, their transition into the pore state did occur with a certain probability depending on the mixing ratio with WT subunits, which increased in step with the fraction of WT subunits. The prepore-to-pore transition under excessive force can be explained by both models similar to the transition without the force. In the E–K pair model, since ~ 5 subunits are within the locus where excessive force was applied, these subunits contain consecutive WT subunits according to their overall fraction. Because the formation of E-K pairs facilitates the release of TMH regions, applying excessive force forces the freely moving regions closer to the membrane, converting them into incomplete β-strands. As discussed above, unfolded *α*-helices in an adjacent mutant subunit are then pulled downwards by the interactions between TMH regions, causing the prepore-to-pore transition of the mutant subunit. In this chain reaction, the application of excessive force at a specific locus is the initiation switch.

Under the lipid model, the WT subunit in the locus is also assumed to be the starting point. The locus contains *N*_x_ × *N*_y_ pixels, where *N*_x_ and *N*_y_ =  ~ 8. When excessive force is applied, the cantilever tip stays for a period, *t*_p_ on one pixel. While the tip moves along one line, a subunit is covered by ~ 4 pixels. This procedure was repeated for *N*_y_ lines. Here, assuming the structural change caused by applying excessive force is reversed after a short period (4*t*_p_), a trial where the force is applied for that period is repeated *N*_y_ times. Using the values of *A* = 10^7^/s, *t*_p_ = 10 μs, and Eqs. 1, 2, 3, and 6 fitting of the pore formation probability yields Δ*G*_0_–*F*_tip_ *x*_β_ = 7.4 ± 0.4 kcal/mol and *F*_mem1_ = 34.2 pN. Assuming Δ*G*_0_ = 14.5 kcal/mol, *F*_tip_ = 99.8 pN, which is close to the applied force value.

Under the E–K pair model, the prepore-to-pore transition induced by applying excessive force could not be quantitatively explained because it is difficult to estimate the number of subunits forming the E–K pairs within a specific locus. Therefore, the lipid model better explains the experimental results but does not completely negate the E–K pair model. Future work will require experimental evaluation of the energy barrier using biochemical and biophysical methods with mutants that can be used to quantify the degree of stabilization between D3 and D1/2.

When a WT subunit in the prepore state was bound to the membrane, membrane protrusion occurred at the central pore (Fig. S1 and S2). This observation suggests that such protrusion is caused by interactions between TMH regions and the membrane, triggering a subunit’s transition under the lipid model. Because the body of D3 is believed to bind and unbind from D2 repeatedly, parts of the TMH regions may contact the membrane surface. During this time, part of the TMH region is converted into β-strands that interact with the membrane and pull the lipid molecules upwards. This interaction may also explain why the height of WT subunits in the prepore state is lower than that of mutant subunits (Fig. S6). However, the lower height might also be caused by the tilt of a subunit through the intersubunit interaction. Furthermore, the height of membrane protrusion decreased to 0.4 nm after the prepore-to-pore transition. This is because the membrane in the central pore is pulled down when the TMH regions insert into the membrane (Fig. 6b). Incidentally, membrane protrusion in the prepore state may be caused by the accumulation of cholesterol and phase separation. However, such a possibility is unlikely because the height of the mutant subunit’s central pore in the prepore state is low.

Previous studies have suggested that PFPs induce changes in the membrane, where SLO causes blebs by direct physical action on the membrane (Keyel et al. 2011). γ-hemolysin was found to form oligomer clusters, inducing a curvature in the membrane (Alessandrini et al. 2013). Moreover, with the CDC suilysin and LLO, lipid micelles or fragments were ejected from the membrane, which affects the membrane diffusion (Leung et al. 2014; Ponmalar et al. 2021). At the low concentration of the CDC, the ejection by ring-shaped oligomers enhances membrane fluidity due to the increased free area per lipid and higher lipid disorder. However, when the concentration is increased and arc-shaped oligomers in the pore state dominate, the lipid ejection is inefficient, and the associated crowding of oligomers results in reduced lipid diffusion.

In this study, the prepore-to-pore transition of hybrid oligomers consisting of WT SLO and mutant subunits was confirmed. My results show that when a subunit is inserted into the membrane, the insertion of an adjacent subunit follows, and this insertion propagates along an oligomer in a chain reaction. Similarly, I observed the propagation of prepore-to-pore transition by applying excessive force. The chain reaction process represents the first direct observation of the subunit collaboration, which is essential for pore formation. During this reaction, subunits are pulled toward the membrane by interactions between TMH–TMH and TMH–lipid molecules, which are a key collaborative factor. In addition, analysis of the chain reaction enabled me to estimate free energies and forces that trigger the prepore-to-pore transition of a subunit and compare two molecular models of the prepore-to-pore transition. This study highlights future research avenues in which further investigations into structural changes of the TMH regions and the TMH–D1/2 interface are required to elucidate the prepore-to-pore transition mechanism in greater detail.

## Materials and Methods

### Expression and Purification

First, I amplified a gene fragment which encodes residues L78-K571 by polymerase chain reaction using Genomic DNA from *Streptococcus pyogenes* Strain SF370; M1 GAS (ATCC). The gene was introduced into pQE30 by In-Fusion Cloning HD Kit (Takara Bio USA, Inc.). The primers used were 5′-GCATGCGAGCTCGGTGCTCCCAAAGAAATGCCAC-3′, 5′-AGGTCGACCCGGGGTCTACTTATAAGTAATCGAACC-3′, 5′- ACCCCGGGTCGACCTGCA-3′, and 5′-ACCGAGCTCGCATGCGGA-3′. Subsequently, the desired SLO mutant with three mutations, namely, G128C, S261C, and C530A (SLO (C/A)-SS) was prepared by PCR. The primers used were 5'-AGAGAGGCCACTGGCTTAGCTTGGGAA-3' and 5'-GCCAGTGGCCTCTCTAGCCATGATACG-3' for C530A, 5'-TAAAAATTGTGAAACCATTGAAAATT-3' and 5'-GTTTCACAATTTTTAGCAAGTACTTC-3' for G128C, and 5'-TCTAAGTGTCAGATTGAAGCAGCTCTA-3' and 5'-AATCTGACACTTAGAATATACCATTG-3' for S261C.

SLO was expressed in BL21 (DE3) by isopropyl β-D-1-thiogalactopyranoside induction. Next, cells were spun down and resuspended in phosphate-buffered saline (PBS). I purified His-tagged SLO according to a previously described method (Nagamune et al. 2004; Sekiya et al. 2007). The cells were disrupted with a sonicator (Qsonica, LLC.) and centrifuged at 9000 × g for 45 min at 4 °C. The supernatant was applied to a Talon metal affinity resin (Takara Bio USA, Inc.). SLO was eluted with an imidazole gradient at 20 mM, 50 mM, 200 mM, and 500 mM. A solution containing SLO was diluted with 20 mM sodium phosphate pH7.4 and 1 mM EDTA and then applied to a column with sulfopropyl resin. The fractions elutedwith NaCl gradient (0–1 M) was exchanged with PBS, 10% (v/v) glycerol, and 5 mM DTT by means of ultrafiltration (Amicon Ultra-15, 10,000 Da MWCO, Millipore, Inc.). For mutant and WT SLO for the hybrid oligomers, DTT was not included. Finally, the purified SLO was stored at − 80 °C until use.

### Preparation of the Supported Membrane

Small unilamellar vesicles (SUV) were prepared as follows. 1, 2-dioleoyl-*sn*-glycero-3-phosphocholine (DOPC, Avanti Polar Lipids, Inc.) and cholesterol (NACALAI TESQUE, Inc.) were mixed at a 4:6 molar ratio in a glass test tube, then the organic solvent was dried under a stream of N_2_ gas. To ensure full evaporation, the test tube was left in a desiccator under aspirator vacuum for more than 1 h. Next, I added ultra-pure water to the test tube, to a total lipid concentration of 2 mg/mL, and vortexed it. The obtained multilamellar vesicles were frozen at − 80 °C and thawed at room temperature 5 times. To prepare the membrane, I mixed 10 μL of SUV and 40 μL of 40 mM MgCl_2_, placed a drop of the solution on a freshly cleaved mica surface, and incubated this for 30 min in a sealed container at 60 °C. The solution was placed at room temperature and, after a while, rinsed with water and Buffer A (20 mM Hepes–NaOH (pH 7.4), 30 mM NaCl and 4–10 mM DTT) or Buffer B (20 mM Hepes (pH 7.4), 30 mM NaCl).

### Visualization of SLO by HS-AFM

I observed pore formation by SLO using an HS-AFM setup described previously (Kodera et al. 2010; Uchihashi et al. 2012). I observed the pore formation process in the membrane via two methods: First, using HS-AFM, I observed the membrane surface, adding SLO into the AFM liquid cell to a final concentration of 0.2 μM. Secondly, 2.5 μL of 0.4–2.0 μM SLO was added to the membrane, and unattached SLO was washed off after ~ 3 min with Buffer A or B several times before observation. The membrane surface was imaged in a 60 μL solution of Buffer A or B with a cantilever (BL-AC10DS-A2 or BL-AC7DS-KU4, Olympus). The cantilever had a resonant frequency of ∼1 MHz in water and a spring constant of 0.1–0.2 N/m. In addition, I used the “interactive imaging mode,” allowing me to add the desired signal to the Z-piezo driver, and a controlled strong tapping force could be applied to a targeted locus of the sample during imaging. The locus consists of *N*_*x*_ × *N*_*y*_ pixels, with *N*_*x*_ and *N*_*y*_ of ~ 8 and an area of ~ 100 nm^2^. The mean force applied for this process was estimated to be 25–100 pN by the following equation:

7 $$\langle F\rangle = \frac{{k}_{\mathrm{c}}\times \sqrt{{{A}_{0}}^{2}-{{A}_{\mathrm{s}}}^{2}}}{2{Q}_{\mathrm{c}}}$$

where *k*_c_ is a spring constant, *A*_o_ is cantilever free oscillation amplitude (~ 1 nm), *A*_s_ is set point amplitude (~ 0 nm), and *Q*_c_ is the quality factor (1 ~ 2). As noted in the discussion, the force was applied for the period, *N*_*x*_ × *t*_p_, per line and repeated *N*_*y*_ times.

### Simulation of the Probability with N Consecutive WT in All Oligomers

I simulated the probability with *N* consecutive WT subunits in all oligomers using Microsoft Excel Visual Basic for Applications with random numbers and the number of subunits set to 30. If the WT subunit is 1 and the mutant subunit is 0, I determined the frequency of *N* consecutive 1’s present in 300 random 0/1 sequences 30 numbers long. This process was repeated five times, and the average was taken as the final probability.

### Fitting of the Probability of the Prepore-to-Pore Transition of Hybrid Oligomers

Fitting was performed in Origin software (OriginLab Corporation) with the equations described above. When fitting the probability of pore formation under excessive force, the probability was divided by *N*_y_ and the result was used as *P*_pore_.

## Supplementary Information

Below is the link to the electronic supplementary material.

Supplementary file1 (MOV 43 kb)

Supplementary file2 (MOV 107 kb)

Supplementary file3 (MOV 213 kb)

Supplementary file4 (MOV 307 kb)

Supplementary file5 (MOV 76 kb)

Supplementary file6 (MOV 182 kb)

Supplementary file7 (MOV 114 kb)

Supplementary file8 (PDF 796 kb)

## Data Availability

The datasets generated during and analysed during the current study are available from the corresponding author on reasonable request.

## Abbreviations

PFPs: Pore-forming proteins
SLO: Streptolysin O
D3: Domain 3
TMHs: Transmembrane β-hairpins
HS-AFM: High-speed atomic force microscopy
WT: Wildtype
CDCs: Cholesterol-dependent cytolysins
PFO: Perfringolysin O
CRM: Cholesterol recognition/binding motif
DTT: Dithiothreitol
PBS: Phosphate-buffered saline
SUV: Small unilamellar vesicles
