# Supplementary material for: Visualizing the Domino-Like Prepore-to-Pore Transition of Streptolysin O by High-Speed AFM
Source: J Membr Biol. 2022 Aug 18;256(1):91–103. doi: 10.1007/s00232-022-00261-x (PMC9884259; doi:10.1007/s00232-022-00261-x)
Supplement: Supplementary file 8 — Supplementary file8 (PDF 796 kb) [file 232_2022_261_MOESM8_ESM.pdf]

Supplementary Information

Visualizing the domino-like prepore-to-pore transition of Streptolysin O by high-speed AFM

*Hirotaka Ariyama †**

†Nano Life Science Institute (WPI-Nano LSI), Kanazawa University, Kakuma-machi, Kanazawa, Ishikawa 920-1192, Japan

Correspondence to: Hirotaka Ariyama

Postal address: Nano Life Science Institute (WPI-Nano LSI), Kanazawa University, Kakuma-machi, Kanazawa, Ishikawa 920-1192, Japan

Tel: +81(0)76-234-4550

E-mail: HirotakaAriyama@outlook.jp

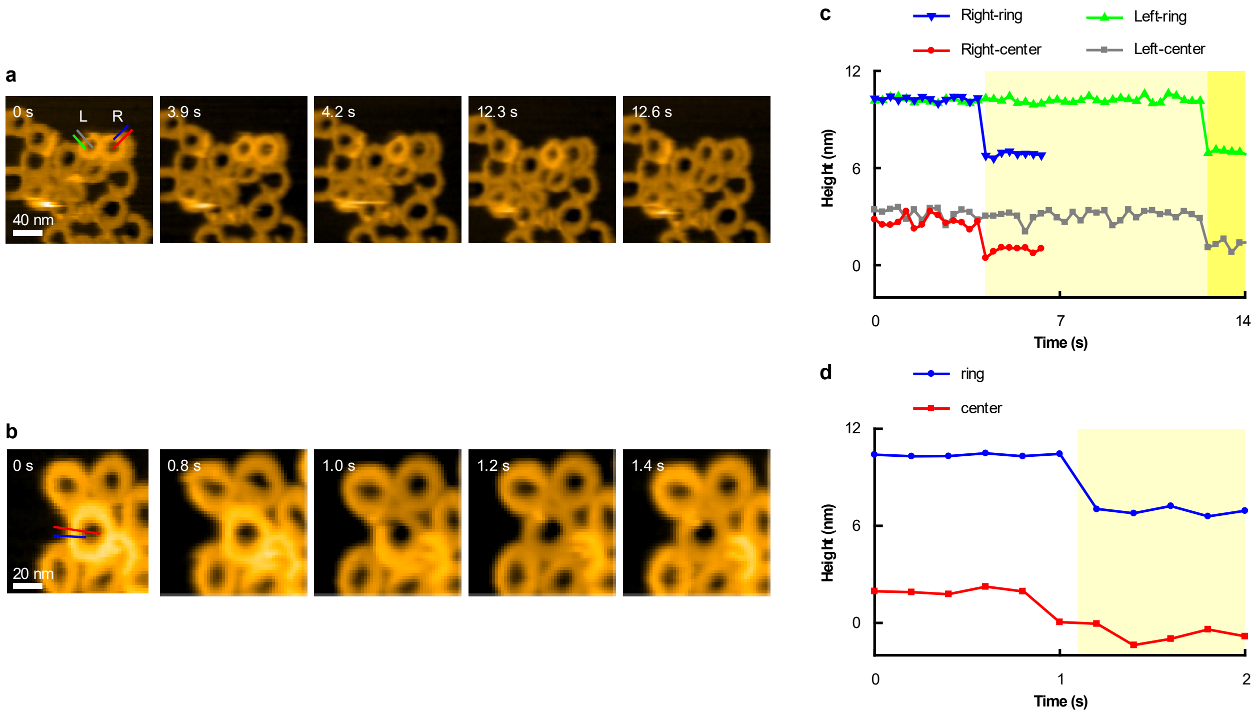

Fig. S1 The height change of the central pore during the prepore-to-pore transition of ring-shaped oligomers.

a, b Successive AFM images of ring-shaped oligomers in the prepore-to-pore transition.

c The height of the central pore and the ring-shaped oligomers during the prepore-to-pore transition over time in (a). “Left-center” represents the height of the central pore of oligomer “L” in (a) from the membrane. “Right-center” represents the height of oligomer “R”. “Left-ring” represents height of “L” and “Right-ring” represents “R.” The height of the central pore of the two oligomers changed from 3 nm to 1 nm simultaneously with the prepore-to-pore transition. Colored regions represent the pore state.

d The height of the central pore and the ring-shaped oligomers during the prepore-to-pore transition over time in (b). The height of central pores decreased from 2 nm to −1 nm in 0.2 s

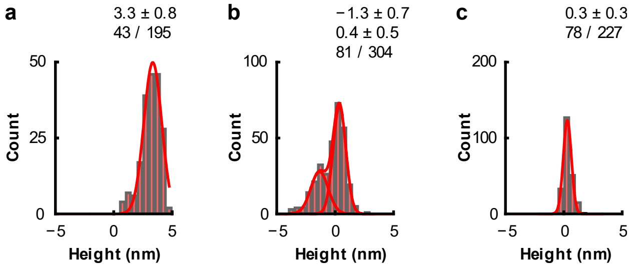

Fig. S2 The height distribution of the central pore of the ring-shaped oligomers from the membrane.

a The height distribution of the central pore of the WT oligomers in the prepore state.

b The height distribution of the central pore of the WT oligomers in the pore state.

c The height distribution of the central pore of the mutant oligomers in the prepore state. The red line indicates Gaussian fitting. The height is represented in mean ± SD. The height, the numbers of oligomers, and the number of frames analyzed are presented at each panel's upper right corner

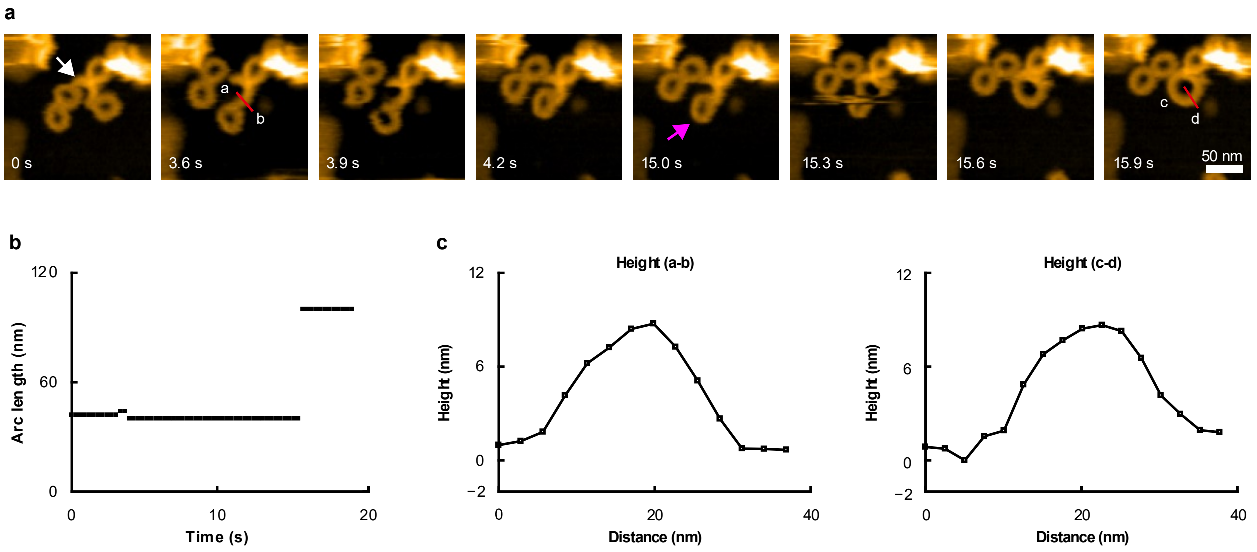

Fig. S3 The formation of a high-order oligomer in the pore state, the change of arc length of the oligomer, and the surface profiles of oligomers before and after formation.

a Successive AFM images showing the formation of a high-order oligomer in the pore state. SLO was added at the final concentration of 0.8 μM; then, unattached SLO was washed away. An arc-shaped oligomer (white arrow) was bound to an oligomer (magenta arrow) at 15.3 s.

b The time course of the length of the arc-shaped oligomer (white arrow in (a)). The arc length increased rapidly when the oligomers formed the high-order oligomer. Subunits bound to other subunits together simultaneously. Only one subunit did not bind to other subunits one-by-one.

c The surface profile of oligomers along the red line in (a) before and after the formation of the high-order oligomer; the height remained constant

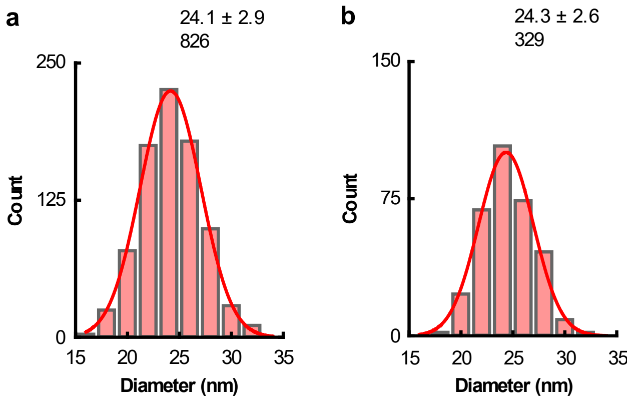

Fig. S4 The change in the diameter distribution of ring-shaped oligomers in the pore state over time.

SLO was added at the final concentration of 0.8 μM, and unattached SLO was washed away. Then, the ring-shaped oligomers were observed at 5 min and 25 min were analyzed.

a The diameter distribution of ring-shaped oligomers at 5 min.

b The diameter distribution of ring-shaped oligomers at 25 min. The red line indicates Gaussian fitting. The numbers in the upper right of the figure represent the diameter as mean ± SD and the numbers of oligomers

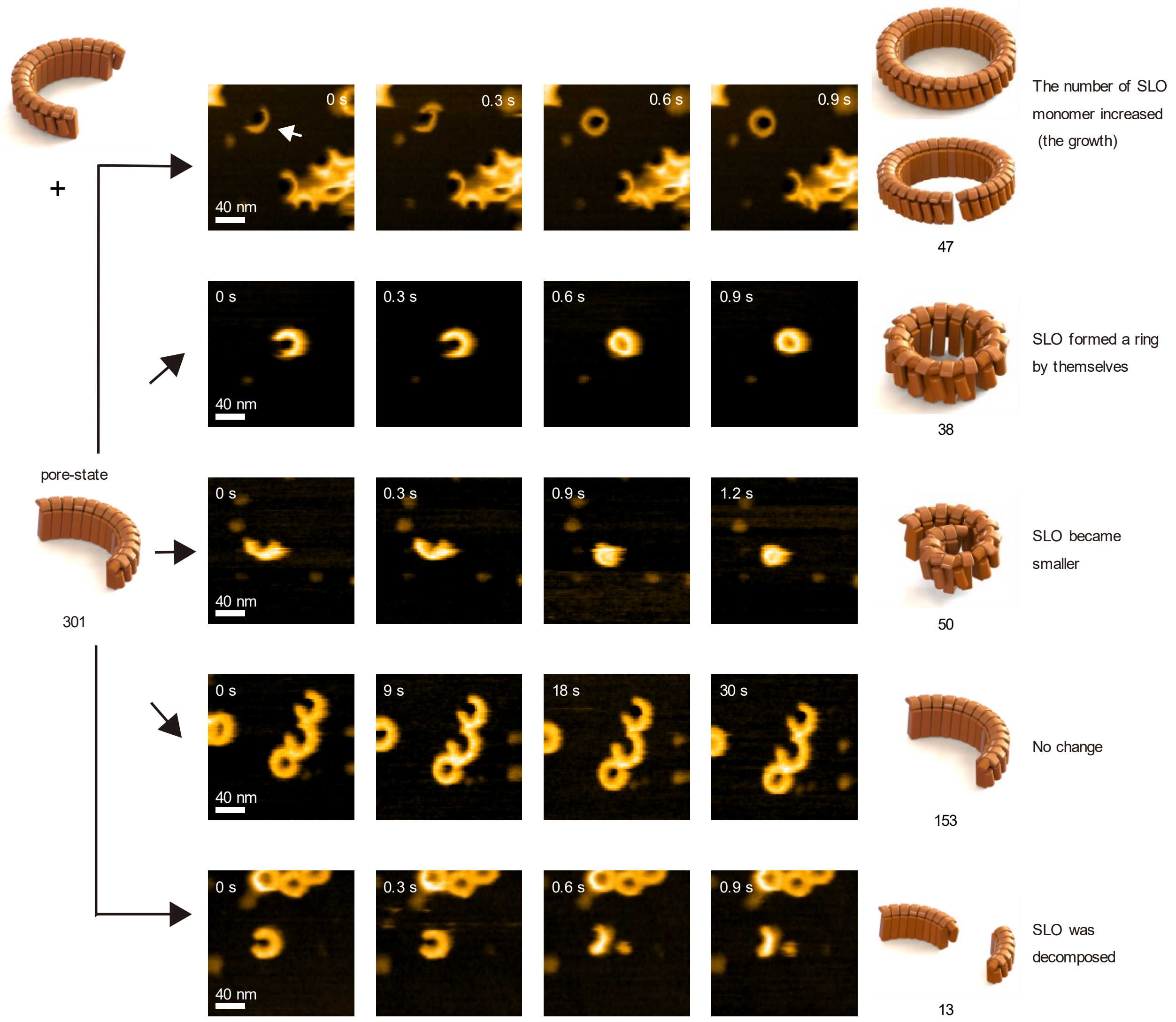

Fig. S5 The classification of shape changes of oligomers in the pore state.

WT SLO was added to the membrane at the final concentration of 0.4–2.0 μM; then, unattached SLO was washed away. The observation of the SLO using HS-AFM revealed that most oligomers were in the pore state. The arc-shaped oligomers in the pore state changed to several shapes, such as increasing in size, forming a ring by themselves, becoming smaller, undergoing no change, or becoming decomposed. The numbers on the bottom indicate the number of oligomers observed

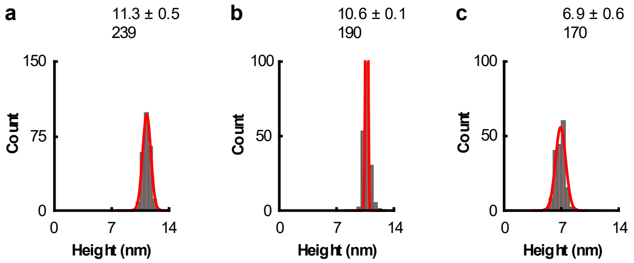

Fig. S6 The height distribution of oligomers in the prepore and pore states on the membrane.

a The height distribution of the mutant oligomers in the prepore state.

b The height distribution of the WT oligomers in the prepore state.

c The height distribution of the WT oligomers in the pore state. The red line indicates Gaussian fitting. The numbers in the upper right of the figure represent the height (mean ± SD) and the numbers of oligomers observed

a

W/M	the number of consecutive WT						
	2	3	4	5	6	7	8
0/10	0.00 ± 0.00	0.00 ± 0.00	0.00 ± 0.00	0.00 ± 0.00	0.00 ± 0.00	0.00 ± 0.00	0.00 ± 0.00
2/8	0.76 ± 0.02	0.13 ± 0.02	0.01 ± 0.01	0.00 ± 0.00	0.00 ± 0.00	0.00 ± 0.00	0.00 ± 0.00
4/6	1.00 ± 0.00	0.83 ± 0.02	0.36 ± 0.01	0.14 ± 0.03	0.05 ± 0.02	0.01 ± 0.00	0.00 ± 0.00
5/5	1.00 ± 0.00	0.98 ± 0.01	0.72 ± 0.02	0.38 ± 0.02	0.19 ± 0.02	0.06 ± 0.02	0.02 ± 0.01
6/4	1.00 ± 0.00	1.00 ± 0.00	0.95 ± 0.01	0.72 ± 0.02	0.44 ± 0.02	0.25 ± 0.02	0.13 ± 0.02
8/2	1.00 ± 0.00	1.00 ± 0.00	1.00 ± 0.00	1.00 ± 0.00	1.00 ± 0.00	0.95 ± 0.01	0.88 ± 0.03
10/2	1.00 ± 0.00	1.00 ± 0.00	1.00 ± 0.00	1.00 ± 0.00	1.00 ± 0.00	1.00 ± 0.00	1.00 ± 0.00

b

p	0.01	0.01	0.001	0.001
the number of consecutive WT	5	6	5	6
$\Delta G_{E\text{-}K}$ (kcal/mol) 10 min	6.57	6.57	6.81	6.81
$\Delta G_{E\text{-}K}$ per subunit (kcal/mol) 10 min	1.31	1.10	1.36	1.14
$\Delta G_{E\text{-}K}$ (kcal/mol) 25 min	6.03	6.03	6.27	6.27
$\Delta G_{E\text{-}K}$ per subunit (kcal/mol) 25 min	1.21	1.00	1.25	1.04

Table S1 The probability of having *N* consecutive WT subunits in all oligomers and the free energy by the formation of an E–K pair.

a The simulated probability of having *N* consecutive WT subunits in hybrid oligomers (P_{con})

b The free energy contributed by an E–K pair is determined based on the probability of pore formation by hybrid oligomers. $\Delta G_{E–K}$ was estimated using data at 10 min and 25 min, and p is the second term in Eq. 2 (1−exp(−kt)), which was set to 0.1 and 0.01

Movie S1 High-speed AFM movie showing the prepore-to-pore transition.

An oligomer simultaneously transitioned into the pore state. In the AFM liquid cell, SLO was added to a final concentration of 0.2 μM. The dynamic process was filmed at 200 ms frame^{-1} (5frame^{-1}) and the obtained movies are played at 5 frames s^{-1}. Scan area, 160× 160 nm ^{2} with 80 × 80 pixels

Movie S2 High-speed AFM movie showing the prepore-to-pore transition.

The prepore-to-pore transition propagated along an oligomer. In the AFM liquid cell, SLO was added to a final concentration of 0.2 μM. The dynamic process was filmed at 200 ms frame^{-1} (5 frame^{-1}) and the obtained movies are played at 5 frames s^{-1}. Scan area, 160 × 160 nm ^{2} with 80 × 80 pixels

Movie S3 High-speed AFM movie showing the formation of a high-order oligomer.

SLO was added at the final concentration of 0.8 μM; then, unattached SLO was washed away. The dynamic process was filmed at 300 ms frame^{-1} (3.3 frame^{-1}) and the obtained movies are played at 10 frames s^{-1}. Scan area, 200 × 200 nm ^{2} with 100 × 100 pixels

Movie S4 High-speed AFM movie showing the prepore-to-pore transition of mutant oligomers with the addition of DTT.

SLO was added at the final concentration of 0.8 μM; then, unattached SLO was washed away. Then, DTT was added to the AFM liquid cell at a final concentration of 4–10 mM. The dynamic

process was filmed at 200 ms frame^{-1} (5 frame^{-1}) and the obtained movies are played at 5 frames s^{-1}. Scan area, 120 × 120 nm^{2} with 100 × 100 pixels

Movie S5 High-speed AFM movie showing the prepore-to-pore transition of hybrid oligomers with a mixing ratio of 4:6 (WT:mutant).

SLO was added at the final concentration of 0.8 μM; then, unattached SLO was washed away. The dynamic process was filmed at 200 ms frame^{-1} (5 frame^{-1}) and the obtained movies are played at 5 frames s^{-1}. Scan area, 120 × 120 nm^{2} with 100 × 100 pixels

Movie S6 High-speed AFM movie showing the prepore-to-pore transition of hybrid oligomers with a mixing ratio of 8:2 (WT:mutant) by applying an external force.

An oligomer simultaneously transitioned into the pore state. SLO was added at the final concentration of 1.2 μM; then, unattached SLO was washed away. The dynamic process was filmed at 200 ms frame^{-1} (5 frame^{-1}) and the obtained movies are played at 5 frames s^{-1}. Scan area, 120 × 120 nm^{2} with 100 × 100 pixels

Movie S7 High-speed AFM movie showing the prepore-to-pore transition of hybrid oligomers with a mixing ratio of 8:2 (WT:mutant) by applying an external force.

The prepore-to-pore transition propagated along an oligomer. SLO was added at the final concentration of 1.2 μM; then, unattached SLO was washed away. The dynamic process was filmed at 200 ms frame^{-1} (5 frame^{-1}) and the obtained movies are played at 5 frames s^{-1}. Scan area, 120 × 120 nm^{2} with 100 × 100 pixels
